# Regulation of integrin and growth factor signaling in biomaterials for osteodifferentiation

**DOI:** 10.3762/bjoc.11.87

**Published:** 2015-05-13

**Authors:** Qiang Wei, Theresa L M Pohl, Anja Seckinger, Joachim P Spatz, Elisabetta A Cavalcanti-Adam

**Affiliations:** 1 Department of Biophysical Chemistry, Institute for Physical Chemistry, University of Heidelberg, INF 253, 69120 Heidelberg, Germany; 2 Department of New Materials and Biosystems, Max-Planck Institute for Intelligent Systems, Stuttgart, Germany; 3 Department of Internal Medicine V, Oncology, Hematology, and Rheumatology, Heidelberg University Hospital, 69120 Heidelberg, Germany

**Keywords:** biomaterials, growth factor, integrin, osteodifferentiation, stem cells

## Abstract

Stem cells respond to the microenvironment (niche) they are located in. Under natural conditions, the extracellular matrix (ECM) is the essential component the in stem cell niche, in which both integrin ligands and growth factors are important regulators to directly or indirectly modulate the cell behavior. In this review, we summarize the current knowledge about the potential of integrin ligands and growth factors to induce osteogenic differentiation of stem cells, and discuss the signaling pathways that are initiated by both individual and cooperative parameters. The joint effect of integrin ligands and growth factors is highlighted as the multivalent interactions for bone therapy.

## Review

### Introduction

Current bone grafting therapeutics do not provide satisfying solutions to the problems of non-healing bone defects. The gold-standard therapy is the grafting of autologous bone; however, it is limited by low availability as well as donor site pain and morbidity on the one hand. On the other hand, the allografts are suffering risk from possible infections and immune response [1]. More recently, stem cell therapy has been extensively studied and gained much focus for bone regeneration to achieve a suitable alternative to current grafting solutions in modern medicine [2].

**Figure 1:**
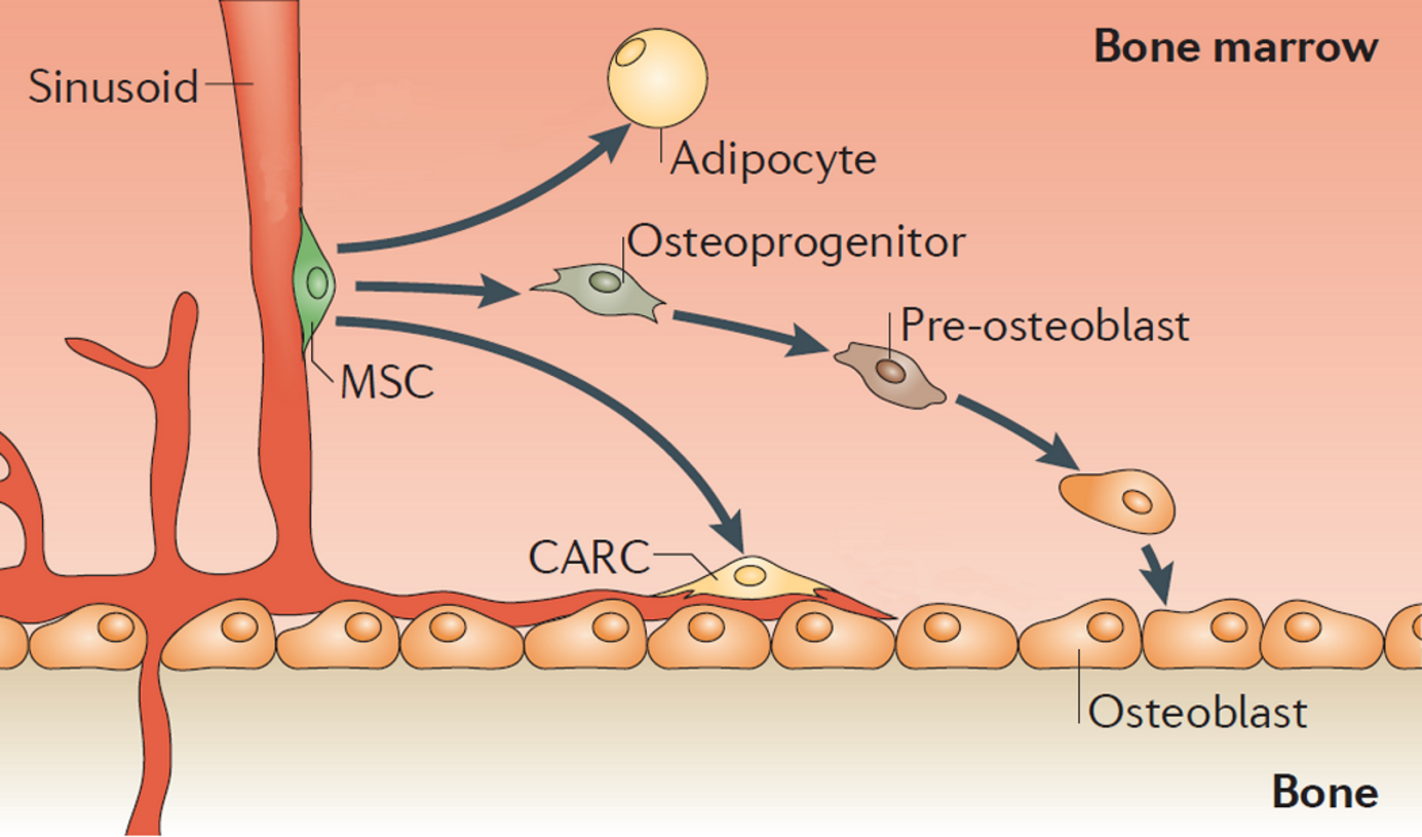
Differentiation potential of mesenchymal stem cells (MSCs) in bone marrow. MSCs can differentiate into a variety of lineages, including osteoblasts, chondrocytes, adipocytes, and reticular cells. Osteogenic differentiation is especially valuable in regenerative medicine approaches. Reprinted with permission from [3]. Copyright 2011 Nature Publishing Group.

Stem cells can differentiate into specialized cells and have self-renewal ability to further generate more stem cells. For example, mesenchymal stem cells (MSCs) derived from bone marrow, can differentiate into a variety of lineages, including osteoblasts, chondrocytes, adipocytes, and reticular cells (Figure 1) [3]. Osteogenic differentiation is especially valuable in regenerative medicine approaches [4]. It has been proven that stem cell fate can be regulated from the specific microenvironment known as stem cell niche. The extracellular matrix (ECM), which virtually all cells in the body are exposed to and stem cells reside in, is an essential component in the stem cell niche [3]. The ECM is not an inert scaffold; instead, it is a dynamic network of molecules secreted by cells. Moreover, its biochemical, biophysical, and mechanical properties have emerged as important regulators for the direct or indirect modulation of cell behavior [4]. Cells interact with the ECM via several kinds of transmembrane receptors, in which the major class involved is integrin’s [5]. Integrin ligands in the ECM include fibronectin, vitronectin, collagen, and laminin, which contain integrin-binding motifs [6]. These integrin-ECM interactions allow cells to sense matrix properties, such as topography and forces, from the ECM and respond in an appropriate manner [4]. Therefore, the use of integrin ligands to regulate stem cell fate becomes a hot spot of research. Both natural and synthetic integrin ligands were developed to control the interaction between biomaterials and stem cells. The effect of the topography and the distribution of the ligands on cell adhesion, proliferation, and differentiation were intensively studied as well [7].

Besides integrin ligands, growth factors, which can stimulate cell growth and differentiation, have also been employed for bone treatment [8–9]. Growth factors are water soluble proteins embedded in the ECM network mainly via non-covalent interactions with glycosaminoglycanes (GAG) [10]. Therefore, the ECM serves as a reservoir by establishing stable gradients of growth factors to regulate their bioavailability [11]. This matrix-immobilization of the factors might result in long-term binding to cell surface receptors, since the binding affinity of ECM-factors is relatively weak compared to growth factor receptor interactions [8]. Moreover, the factors can be released upon matrix turnover and degradation.

It has been proven that a large number of growth factors can induce bone healing [9], for example, bone morphogenetic proteins (BMPs) [12], transforming growth factor beta (TGF-β) [13], fibroblast growth factors (FGFs) [14], vascular endothelial growth factor (VEGF) [15], etc. Among them, BMPs are believed to be the most effective growth factors to induce bone growth [9]. However, when the BMP doses used clinically are much higher than the physiological concentrations, e.g., in the case of a systemic stimulation way, they lead to high costs of treatment and side-effects like pathologic changes or ectopic ossification [1]. To solve this problem, local delivery concepts that use implantable devices have been widely investigated [8–9].

Integrin ligands and growth factors are not independent systems for modulating osteogenic differentiation. It has been shown that integrins exert an extensive crosstalk with many growth factor receptors [16]. Integrin ligands actively participate in the regulation of growth factor-mediated signaling. Ligand–integrin interactions can induce ligand-independent partial activation of growth factor receptors and result in optimal cell survival and migration signals. Growth factor-mediated activation of the receptors leads to clustering of integrins and activation of integrin signaling [8,17]. In a word, the crosstalk between integrins and growth factor receptors is bidirectional that integrins may affect receptor signaling, and receptors may regulate integrin expression and activation [16].

In the first part of the review, we summarize how integrin ligands control cell adhesions and provide insight on how these interactions can regulate stem cell fate. In the second part, we report the current knowledge about growth factors and their ability to induce osteogenic differentiation of stem cells and we outline the delivery of these factors in vivo and in vitro. Furthermore, the studies on the cooperation of integrin ligands and growth factors for bone therapy are reviewed, and the coordinated signaling of integrins and growth factor receptors are discussed.

### Integrin ligands for cell adhesion and stem cell fate

In order to enhance the effectiveness of cell-based bone therapy, it is important to understand the signals from integrin–ligand interactions. New technologies have been employed to provide insights into how cells sense the information from ligands and how they respond at the molecular level, which ultimately regulate the differentiation of stem cells.

#### Integrin and integrin ligands

**Figure 2:**
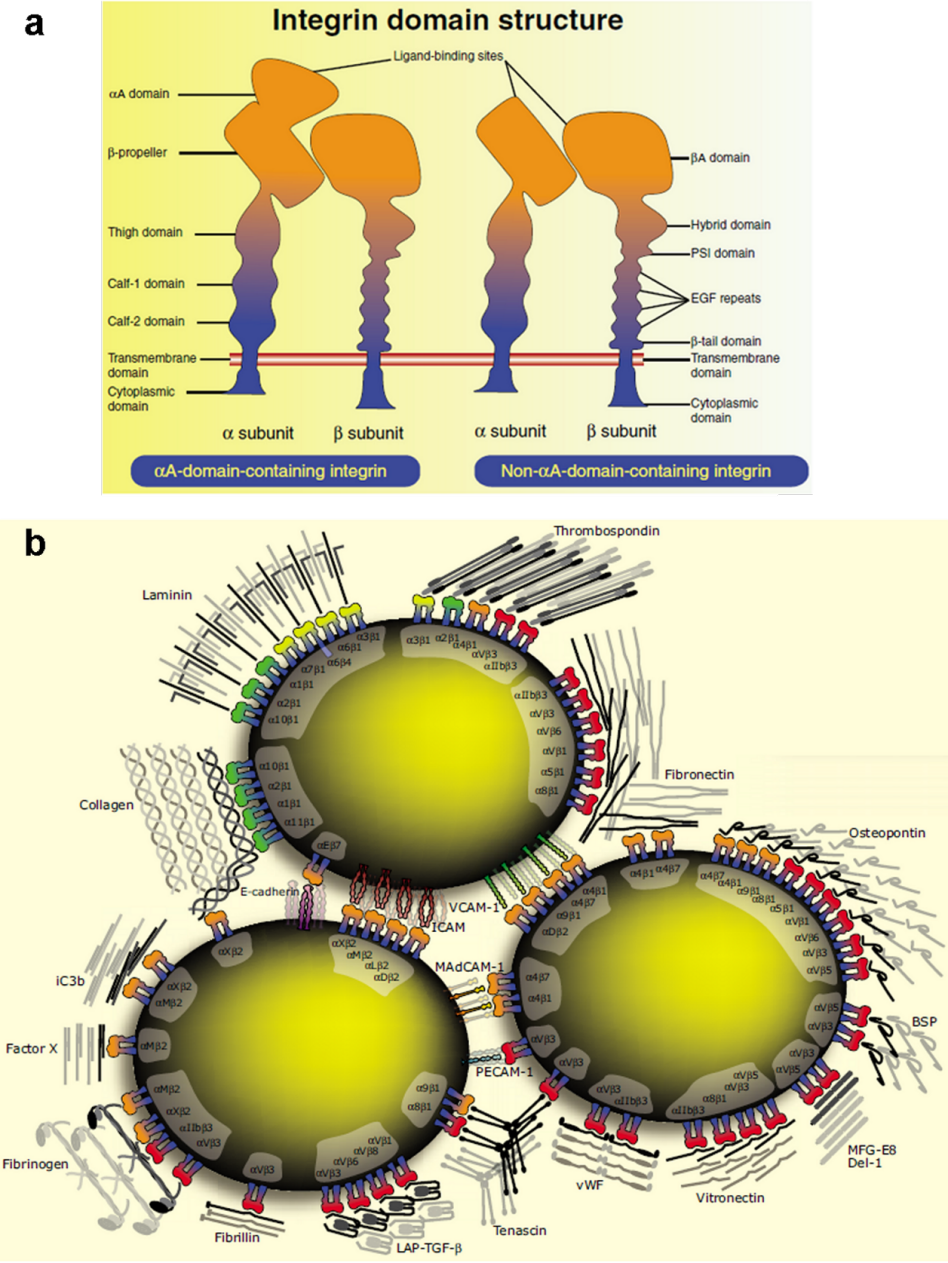
(a) The structure of the integrin heterodimeric receptors with α and β subunits. (b) The major integrin–ligand combinations on the cell surfaces. Abbreviations: BSP, bone sialoprotein; Del-1, developmental endothelial locus-1; EGF, epidermal growth factor; ICAM, intercellular cell adhesion molecule; iC3b, inactivated complement component C3b; LAP-TGF-β, latency associated peptide transforming growth factor β; MAdCAM-1, mucosal addressin cell adhesion molecule 1; MFG-E8, milk fat globule EGF factor 8; PECAM-1, platelet endothelial cell adhesion molecule 1 (CD31); PSI, plexin/semaphorin/integrin homology; VCAM-1, vascular cell adhesion molecule 1; vWF, von Willebrand factor. Reprinted from [18]. Copyright 2006 The Company of Biologists.

Integrins, which are non-covalently linked heterodimeric transmembrane receptors, contain an α and a β subunit. Both subunits exhibit mostly short cytoplasmic domains and large extracellular domains (Figure 2a). The cytoplasmic domains coordinate the assembly of cytoskeletal proteins and signaling complexes, while the extracellular domains engage either ECM components or counter receptors of the adjacent cells [18]. Therefore, the integrins serve to link the two compartments, namely the ECM and the intracellular actin filamentous cytoskeleton across the plasma membrane. The interactions between integrins and ligands result in two major functions. First, the interactions physically integrate the ECM-bound cells and their cytoskeleton. Second, the signals resulting from these interactions enable cells to sense the chemical and mechanical properties of the microenvironment (niche) and to respond by activating signaling systems for regulating the cell fate [19]. Conversely, the contraction of the attached cytoskeleton pulls integrins together into larger adhesive clusters [7].

The type of the integrin–ligand interactions and the integrin–ligand pairs have been well described in previous reviews [18,20]. Most integrin receptors can bind a wide variety of ligands. Many ECM ligands and cell surface adhesion proteins, on the other hand, bind to multiple integrin receptors (Figure 2b) [18]. A set of receptor–ligand combinations with high-affinity interaction has even been identified. The best characterized and most widely used ligand is the arginine-glycine-aspartic acid (RGD) sequence. RGD motifs are present in many ECM glycoproteins, e.g., fibronectin, vitronectin and osteopontin [21], and are recognized by all five α_V_, two β1 (α5 and α8), and αIIbβ3 integrins [18]. More particularly, RGD binds in a pocket between the α and β subunits. The arginine residue (R) fits into a cleft in a β-propeller module in the α subunit, in the meanwhile, the aspartic acid residue (D) coordinates a cation bound in the von Willebrand factor A domain of the β subunit [18].

**Figure 3:**
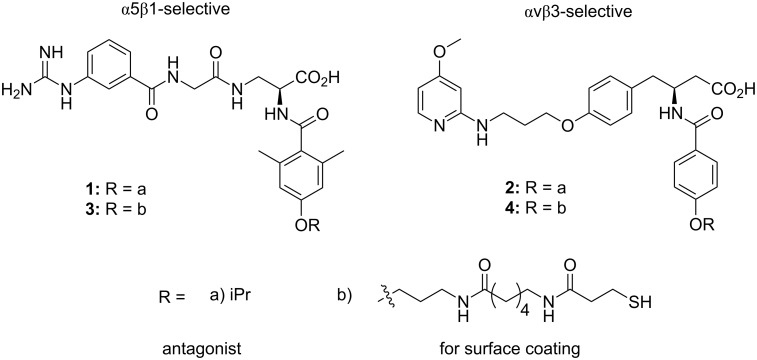
The chemical structure of the α5β1-selective (left) and the αvβ3-selective (right) peptidomimetics. Reprinted with permission from [22]. Copyright 2013 Wiley.

To enhance the selectivity for recognizing distinct integrin subtypes, synthetic specific ligands have been developed [22]. In a recent work, peptidomimetics of the α5β1 antagonist and the αvβ3 antagonist were synthesized, respectively (Figure 3). Both peptidomimetics can selectively mediate cell adhesion by binding with the relative single integrin subtype without losing activity, while avoiding unspecific adhesion and integrin binding. This technology is helpful to understand how cell functions and responses are regulated by a single integrin subtype and is further essential to modulate the osteogenic differentiation of stem cells.

#### Integrin–ligand interactions to regulate cell adhesion and differentiation

Integrin ligands directly control the cell adhesion and spreading to affect the remolding of the cytoskeleton. The response of the cells activates the signaling pathways to regulate stem cell fate. The affinity of integrin–ligand interactions and the density of the ligands affect cell differentiation. MSCs differentiate towards osteoblasts, when they are cultured on high-affinity cyclic RGD immobilized substrates. When cultured on low-affinity linear RGD functional surface, MSCs express myogenic markers at high ligand density and neural markers at low ligand density [23]. In the other cases, when the ligands are efficient enough to induce cell attachment, the concentration and composition of the ligands do not affect cell differentiation; thus, the distribution of the ligands regulates the shape and spreading of the adherent cells [24]. In the case of single human epidermal stem cells, cells initiates terminal differentiation at higher frequency on a small circular adhesive pattern (20 μm diameter) than on a large circular pattern (50 μm diameter) [25]. The authors further revealed that G-actin level is the key to control the cytoskeletal tension. G-actin inhibits serum response factor (SRF) activity by limiting the availability of its co-factor MAL, when cells spread on large pattern. While cell spreading is restricted on small pattern, the level of G-actin is reduced, SRF activity increases and JunB expression is stimulated. In the case of human mesenchymal stem cells, the differentiation program is determined by adhesion and spreading. Spread cells more likely differentiate into osteogenic lineage, and round cells more likely differentiate into adipogenic lineage [26].

**Figure 4:**
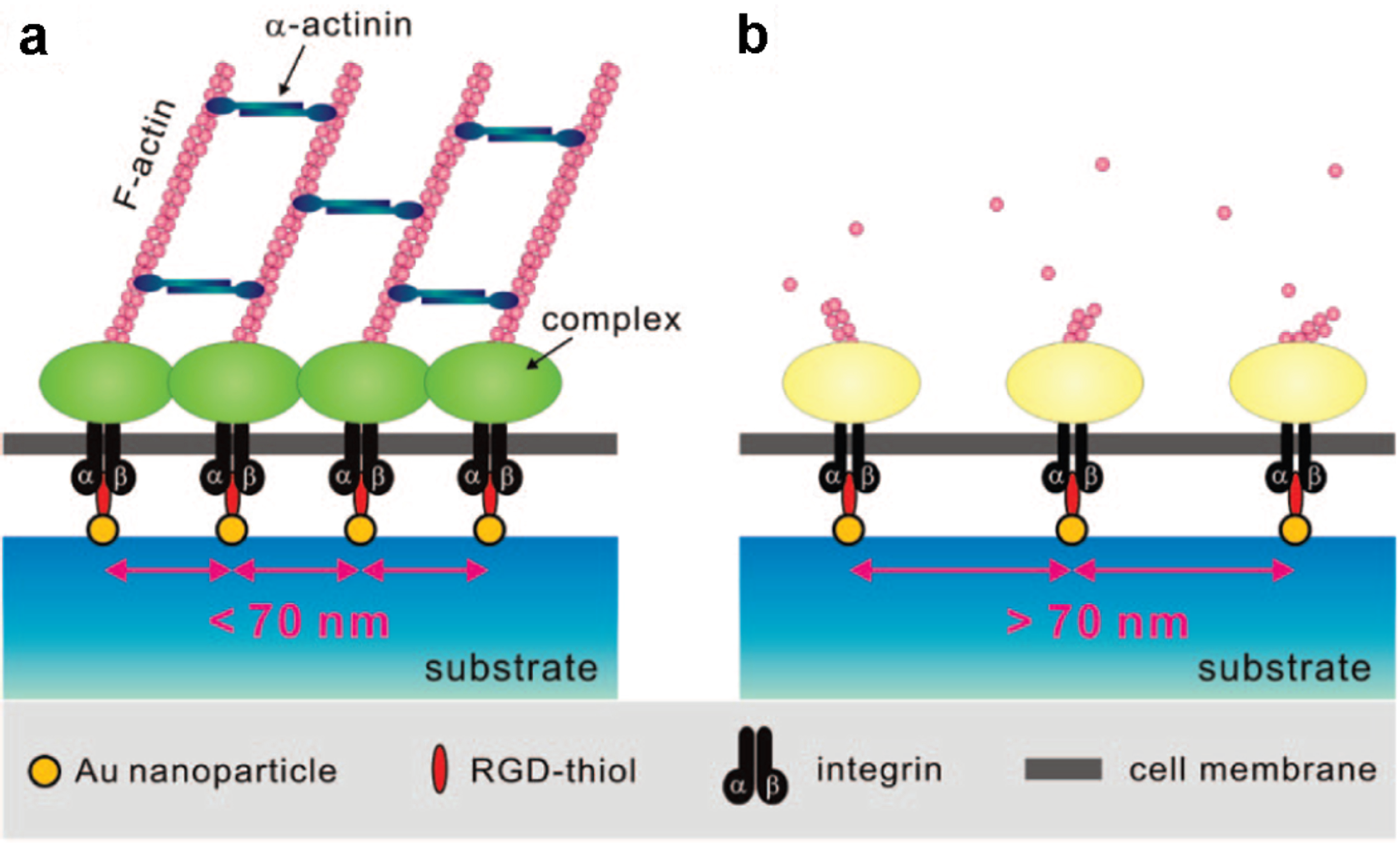
When the distance between two neighboring integrin ligands is <70 nm, the focal adhesions and contractile actin cytoskeletal stress fibres allow cell spreading (a). When the distance is >70 nm, the formation of focal adhesion and actin fibers is hindered (b). Reprinted with permission from [29]. Copyright 2009 American Chemical Society.

To study cell spreading at the molecular level, nanotopography of the ligands available for binding has been modulated. The features of the nanoscale surface have a similar size compared to individual cell receptors, thus it is possible to target receptor-driven pathways and modulate cell responses [7]. Here, the cyclic RGDfK peptides are precisely immobilized on substrates via hexagonally close-packed gold nanodot arrays prepared by block-copolymer micelle nanolithography [27]. The critical distance of the ligands that limited cell spreading is approximately 70 nm (Figure 4). When the distance is larger, the formation of focal adhesions and actin cytoskeletal stress fibers is restricted. As a result, cells are less adhesive on the substrates and turn into quiescent or even apoptotic by anoikis. Contrarily, when the ligands are closer than 70 nm, cells form focal adhesions and contractile actin fibres which enable spreading [27–28].

Similarly, ligand nanotopography is also important to control the spreading of stem cells for further regulating their differentiation. Osteogenic differentiation of MSCs can be enhanced on helical self-assembling nanoribbons with periodic binding sites in every 63 nm. However, when the distance of the periodic binding sites increases to about 100 nm on twisted nanoribbons, an osteoblast commitment cannot be observed [30].

When the ligand nanoarray with a distance just over 70 nm was disordered, the adhesion and spreading of the cells are enhanced [29]. Although the average center-to-center distance of the ligands is kept constant, some ligands can be arranged more densely and others more loosely. The distance of the ligands on the denser parts shall be smaller than 70 nm, thereby allowing integrin clustering and assembly focal adhesions to induce cell spreading. In a similar concept, a controlled nanodisordered pattern, which is not highly ordered but not random either, induces rapid osteogenesis from skeletal stem cells, due to the enhanced cell spreading [31]. Additionally, the well-designed highly ordered nanopatterns might be used to maintain the phenotype of MSCs. These patterns reduce but do not completely inhibit MSC adhesion. Therefore, the differentiation of MSC to both osteogenesis and adipogenesis is limited. As a result, cells are self-renewed without loss of phenotype [32].

In a recent study, how nanoscale clustering of integrin ligands alters the mechano-regulation of integrins has been revealed with the assistance of molecular tension fluorescence microscopy [33]. In the step of nascent adhesion formation, integrin tension driven by actin polymerization is in an average of 1–3 pN per ligand on the nanoarrays with distance both smaller and larger than 70 nm (approximately 50 and 100 nm, respectively). However, in the step of focal adhesion maturation, the tension on different nanoarrays is significantly different. In the 50 nm case, the average tension increases to about 6–8 pN due to the actomyosin-contractility, while in the 100 nm case, the tension even decreases due to the destabilization of integrin clusters. These results agree with the above cell spreading studies, and are important to understanding the mechanotransduction for regulating stem cell fate.

As a reverse process of cell differentiation, integrin adhesion also influences the reprogramming of differentiated cells to pluripotency. In a recent study, fibroblast adhesion is regulated by parallel microgrooves and aligned nanofibres, which significantly improve cell reprogramming [34]. The regulated cell adhesion can decrease histone deacetylase activity and upregulate the expression of WD repeat domain 5 (WDR5). As a result, the mechanomodulation of the epigenetic state of cells can be controlled. Cell reprogramming allows the patients who have a limited number of harvestable stem cells to find new source for bone healing.

#### Signaling mechanisms of integrin–ligand interactions to regulate stem cell fate

The signaling pathways that are implicated in triggering cell differentiation in response to the integrin–ligand interactions have been mapped [7,35]. Generally, integrin–ligand interactions elicit the activation of focal adhesion kinase (FAK) and its downstream target-effectors [36]. FAK is a tyrosine kinase and embedded in focal adhesions, the distribution of which is responsive to cell adhesion and spreading. The integrin–ligand interactions also activate a series of other biochemical signals, such as the Ras-ERK cascade, and PI3-K and Rho family proteins [37]. Another tyrosine kinase Src also appears to be important for the regulation of focal adhesion organization [38]. Both FAK and Src play an important role to regulate G-proteins involved in filopodia, lamellipodia, and contraction [7]. Moreover, FAK can directly serve to gene regulation. It can transfer from focal adhesions to the nucleus to target ubiquitination of the cell-cycle mediator p53 and act as a transcription co-regulator with the GATA4 zinc-finger transcription factor [7,39–40]. Additionally, Rho A kinase (ROCK) can mediate intracellular tension through Rho-driven myosin activation to control the contraction of stress fibres [26,41]. Rho and ROCK have been shown to regulate MSC response to osteogenic niche [42]. The inhibition of ROCK may inhibit MSC growth and differentiation [43].

Integrin–ligand interactions that directly affect the cytoskeletal tension can further alter the shape of the nucleus, chromosomal arrangement and gene transcription. Therefore, the interactions may directly affect cell phenotype [7]. A cell can be described as a mechanical unit rather than biochemical unit in the theory of mechanotransduction. In this theory, integrin–ligand interactions cause cytoskeleton reorganization, which further affects the nuclear morphology, since the nucleus connects to the other side of the cytoskeleton. The change of the nuclear morphology subsequently propagates to the interphase chromosomes which are linked to the nuclear lamins at matrix-attachment regions [44]. Therefore, the genome and gene expression may be influenced. Based on this theory, the MSC differentiation can be modulated by the lamin-A level. Low lamin-A levels result in a more adipogenic differentiation, while the osteogenic differentiation is enhanced by increasing lamin-A levels [45].

### Growth factors for modulating osteogenic differentiation

Growth factors, which can be found in all tissues, are important parameters to regulate a variety of cellular functions. They are able to stimulate or inhibit cell proliferation, migration, differentiation, or even gene expression [46–47]. The very same growth factors might trigger different functions in different cell types, because of their pleiotropic characters [48]. The same factors can even act in opposing manner, depending on the local concentration, to up- or down-regulate the synthesis of receptors. Some growth factors need to bind to ECM components, e.g., collagen or heparin to be stabilized or even to be activated [47,49]. Together with cytokines, growth factors, like bone morphogenetic proteins 2 (BMP-2), are involved in processes like wound healing and the bone regeneration [50–51]. BMP-2, which is locally secreted by skeletal and extraskeletal tissues, is part of the complex bone tissue consisting of different cell types and mineralized ECM. The interplay of these bone-matrix-derived growth factors with other molecules, such as hormones, regulates the differentiation of MSCs into osteogenic lineage [48,52], which results in an extraordinary potential for growth, regeneration and remodeling [50].

#### Bone morphogenetic proteins

BMPs belong to the superfamily of transforming growth factors-beta (TGF-β). Currently there are 14 known BMPs, which form a subfamily together with the growth differentiation factors (GDF) [53]. BMPs were originally known for their ability to induce the formation of de novo bone. However, nowadays they have been identified to affect numerous tissues during development and in the adult, besides influence bone formation and healing [54]. BMPs are involved in versatile non-osteogenic development processes, such as cell proliferation, differentiation, apoptosis, cell fate determination, and morphogenesis of many organs and tissues, gonads and the nervous system [55]. With a few exceptions, the physiological functions of BMP family members are mostly related to bone and cartilage formation as summarized in Table 1. Among those BMP-2, BMP-4, BMP-6, BMP-7, and BMP-9 are known to induce complete bone morphogenesis.

**Table 1:** Overview of the bone morphogenetic protein family. BMP members in humans and their main biological functions [53,56].

BMP	Alternative name	Main physiological function

BMP-2	BMP-2a	Cartilage and bone morphogenesis, heart formation
BMP-3	BMP-3a, Osteogenin	Negative regulator of bone morphogenesis
BMP-3b	GDF-10	Negative regulator of bone morphogenesis
BMP-4	BMP-2b	Cartilage and bone morphogenesis, kidney formation
BMP-5	–	Limb development, bone morphogenesis
BMP-6	Vgr-1, Dvr-6	Hypertrophy of cartilage and bone morphogenesis, oestrogen mediation
BMP-7	OP-1	Cartilage and bone morphogenesis, kidney formation
BMP-8	OP-2	Bone morphogenesis, spermatogenesis
BMP-9	GDF-2	Bone morphogenesis, development of cholinergic neurons, glucose metabolism
BMP-11	GDF-11	Axial skeleton patterning, eye development, pancreas development, kidney formation

**Figure 5:**
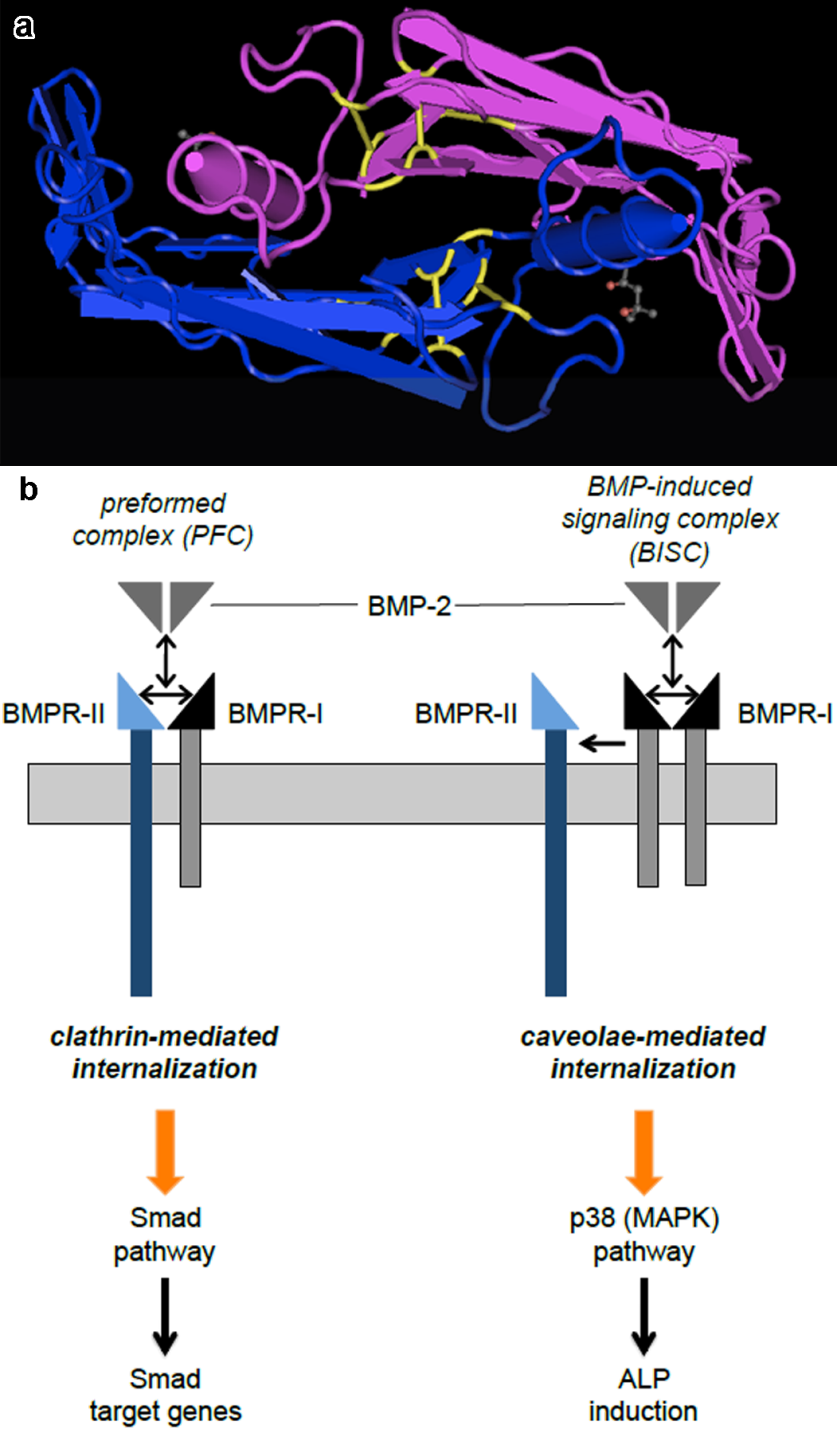
(a) BMP-2 homodimer. 3D-Structure of a BMP-2 homodimer (blue and pink) with cysteine residues, highlighted in yellow to show the intra- and intermolecular disulfide bonds, which determine the three dimensional structure of the protein [59]. (b) Smad dependent and independent BMP signaling pathways. Smad-dependent signaling cascades are induced upon binding of the ligand to a preformed complex (PFC) of BMPR-I and BMPR-II and subsequent internalization via clathrin-mediated internalization. In contrast to that, binding of the ligand to BMPR-I and subsequent recruitment of BMPR-II (BISC) results in caveolae-mediated internalization and triggers Smad independent signaling via p38 (mitogen-activated protein kinase (MAPK)) signaling, resulting in ALP induction. Adapted from [60].

BMPs are transcribed as large precursor proteins composed of a signal peptide, a prodomain and a mature domain. The proproteins dimerize after the signal peptide has been removed and are enzymatically cleaved to yield the biologically active dimeric mature protein [57]. The amino acid sequence of BMPs and their "cystine knot" motif, which is composed of seven cysteine units, is highly conserved [50]. Six of the seven cysteine residues Cys14/Cys79, Cys47/Cys113, and Cys43/Cys111 form intramolecular disulfide bonds to stabilize the monomer, whereas the seventh cysteine (Cys78) contributes to the formation of an intermolecular bond between the two monomers for dimerization. (Figure 5a) [58–59].

This cystine knot, which is highly resistant to heat, denaturants, and extreme acidic pH, defines the three dimensional structure of the protein and thus determines the unique properties of BMPs [47,61–62]. Although homodimers are considered to be the standard form, heterodimers are naturally formed [63]. The heterodimers can be engineered by the co-expression of two different recombinant BMPs. The affinity of the monomers to form dimers for maintaining the cystine knot motif leads to heterodimer formation. This is especially interesting, as BMP-2/BMP-7 for example, shows higher bioactivity compared to their corresponding homodimers [57].

BMP receptors (BMPRs) belong to the group of serine/threonine kinase transmembrane receptors and are subdivided into type I and II receptors [64–65]. There are three type I receptors, namely BMPR-IA (also known as ALK-3, activin receptor-like kinase), BMPR-IB (ALK-6), and the activin receptor ActR-IA (ALK-2); as well as three type II receptors, BMPR-II, ActR-II, and ActR-IIB. The binding of the ligands to these receptors results in heterooligomeric complexes, and thus leads to the activation of signal transduction [57,66–70]. In fact, the binding of BMP can induce different signaling cascades. Either the ligand binds to a preformed complex (PFC) consisting of a type I and II receptor, or the ligand mediates homodimerization of BMPR-I, followed by recruitment of BMPR-II (Figure 5b). The latter oligomerization mode, which is referred to as BMP-induced signaling complex (BISC), leads to internalization via caveolae and induces Smad-independent signaling cascades, resulting in alkaline phosphatase induction through p38 (mitogen-activated protein kinase (MAPK)) signaling cascade. Binding to PFCs triggers clathrin-dependent internalization and initiates a Smad-dependent pathway by phosphorylation of the receptor-regulated Smads (R-Smads, Smad1, 5, or 8) [60,71–72]. After phosphorylation, R-Smads are released from the BMP receptor and form a complex with the common mediator Smad (Co-Smad, Smad 4). This Smad complex translocates into the nucleus and activates the transcription of specific target genes such as the inhibitor of differentiation (Id) (Figure 5b) [70].

#### Growth factors for bone therapy

The demographic challenge of an aging population leads to a clinical as well as a socioeconomic need for repair and regeneration of traumatized or lost tissue. Engineering delivery systems to create cartilage and bone for orthopedic application is therefore a pivotal need [48]. Conventional methods for bone therapy with autologous bone grafts are accompanied by many side effects, e.g., blood loss, risk of infection, and postoperative pain at the autograft site, as well as extended operation times. To solve these problems, local stimulation with growth factors are provided as promising alternatives for bone tissue engineering [73].

Principally, there are two strategies to engineer bone tissue via direct growth factor delivery. Growth factors can be either locally implanted on carrier matrices or systemically distributed. Compared to former case of local delivery, the main advantage of the latter case, systemic stimulation, is that the employed injectable therapeutics is less invasive. However, the disadvantages are apparent as well. Growth factors in service conditions have a markedly shortened half-life and must be administered over long stimulation periods of several days. Moreover, excessive dosage causes side-effects like pathologic changes or ectopic ossification. Therefore, fewer studies have been devoted toward this systemic growth factor delivery [74]. Instead, local delivery concepts that are performed by implantable devices have been widely investigated over the last decades. The well-developed delivery systems for addressing confined bone regeneration include both absorbable and non-absorbable scaffolds, as well as both natural and synthetic materials. Depending on the application site, excipients of different geometries and stiffness were investigated and have shown to affect bone healing [75–76].

Actually, some confined growth factor delivery systems have already been clinically approved, when stimulation is only temporarily necessary until the regeneration occurred [77–78]. However, since bone regeneration is a complex cascade that is regulated by three major components, namely, cells, ECM, and morphogenetic signals, efficient tissue engineering of bone and cartilage must be subjected to each of these parameters [76,79]. A delivery system should therefore ideally fulfill certain requirements. It should be biological and immunological inert; promote specific cell adhesion, proliferation, and angiogenesis; provide growth factors; be rigid to withstand deforming forces (depending on application); be timed biodegradable; neither cause acute nor chronic inflammation; be easily stored and handled (sterilized); and the last be cost-effective [75,80].

**Figure 6:**
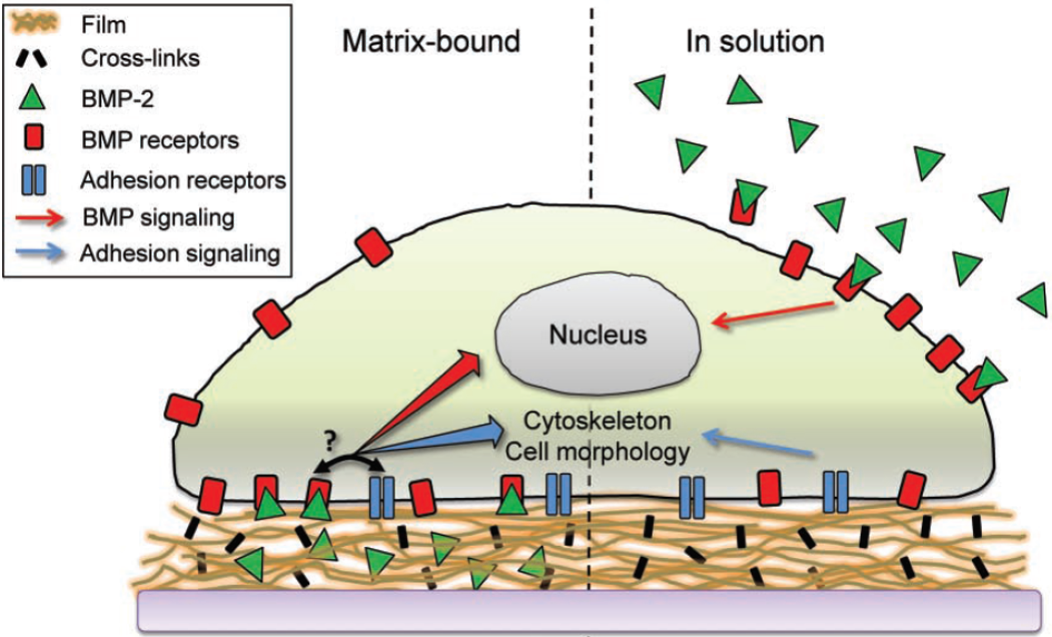
Growth factors, e.g., BMP-2, can be immobilized on the substrates to mimic the matrix-bound form (left), as well as be encapsulated to mimic the soluble form in natural conditions. The different delivery approaches may influence the crosstalk between the growth factors and integrin ligands (discuss below). Reprinted with permission from [85]. Copyright 2011 Wiley.

The present delivery systems and methods have been systematically reviewed in recent literature [8–9,81]. In brief, growth factors in living systems exist in both soluble and matrix-bound forms [82]. Therefore, growth factor delivery can be designed by both encapsulation and surface immobilization approaches (Figure 6). The proteins should be slowly released from the delivery systems in the former case. The latter immobilization systems have the advantage of controlled and sustained influence on cell behavior [82–83], however, the orientation of many growth factors in single molecule level is not well controllable, which decreases the activity of the immobilized factors. In addition, immobilizing osteoinductive proteins on preferably osteoconductive matrices enables not only to control but even to prolong regenerative stimulation, thus minimizing side effects, while augmenting healing.

Moreover, some growth factors, e.g., FGF family members, play an important role in cell reprogramming. FGF2 can promote fibroblast cells to reprogramme to induce pluripotent stem cells (iPSCs) [84]. The reason is that FGF2 sustains extracellular-signal-regulated kinase (ERK) phosphorylation and the expression of pluripotency marker genes, e.g., NANOG. As mentioned in the section about integrin–ligand interactions to regulate cell adhesion and differentiation, cell reprogramming increases the source for bone therapy.

#### Joint effects of integrin ligands and growth factors

Since both, integrin ligands and growth factors play an important role in regulating osteogenic differentiation of stem cells as discussed above, these two parameters have been employed together for developing new biomaterials to enhance bone regeneration. For example, the microspheres with immobilized RGD peptide and adsorbed BMP-2 exhibits high potential for cell adhesion and differentiation of MSCs [86]. In another case, the pro-osteogenic α2β1 integrin-specific GFOGER peptide ligands and BMP-2s are integrated in matrix metalloproteinase (MMP)-degradable PEG-maleimide hydrogels. The peptide ligands successfully host stem cells in vivo, and the sustained release of low doses of BMP-2 direct endogenous stem cell differentiation and promote bone healing [87].

Furthermore, the signal integration between integrins and growth factor receptors has been detected [35], which is in accordance with the positive experimental results on the combined effect of ligands and factors as shown above. Several distinct classes of signal coordination, including concomitant activation, collaborative activation, and direct activation signaling pathways, have been described [88].

First, the integrins and growth factors can activate independent signals to trigger the same signaling molecules as concomitant activation. It has been reported that the Ras-MAPK (mitogen-activated protein kinase) pathway, phosphoinositide 3-kinase (PI3K)-Akt pathway, and regulation of Rho family GTPases can be activated by this concomitant signaling way [35,88–89]. Second, integrin activation assists in growth-factor-dependent receptor signaling, as collaborative activation. Integrins may gather some signaling proteins to create an environment to help some growth factor receptors for their interaction with downstream signaling molecules [90]. These receptors include the epidermal growth factor receptor (EGFR), Met, platelet-derived growth factor receptor (PDGFR), insulin receptor, and vascular endothelial growth factor receptor (VEGFR) [35,88]. The collaboration is important for adhesion-dependent cell survival. Integrin-mediated cell adhesion responds to growth factors. When this response is impaired by cell detachment, it can result in cell growth arrest and even anoikis [91–92]. Third, integrins can also directly activate growth factor receptors by a growth-factor-independent receptor signaling pathway as direct activation. For example, EGFR phosphorylation can be induced by integrins in the absence of EGF [93]. Integrin-induced effects on receptor activation are distinct from the effects that are stimulated by the growth factor alone [88].

The growth factor receptor can activate the integrin gene expression to increase the amount of expressed integrins, which further activate the signaling pathways as mentioned above to amplify the signal [88]. Furthermore, integrins in some conditions can negatively regulate the growth factor receptor signaling. Ligand–integrin interactions have the ability to trigger phosphatase activation and recruitment to inhibit the signaling of growth factor receptors [88].

## Conclusion

It may be insufficient to directly implant cells into human body, which may die or differentiate to the unexpected directions. Therefore, the appropriate extracellular environment must be carefully considered in biomaterial science to employ stem cells for cell therapies. Integrin ligands and growth factors are two of the most important parameters in the stem cell niche to determine the cell fate. In this review we highlighted the effect of integrin ligands and growth factors on the regulation of osteogenic differentiation of stem cells for bone regeneration. These two parameters can be either individually or cooperatively employed to induce cell differentiation. The relationship between these two parameters was also underlined. Although many signaling pathways that initiated by these two have been described, a deeper understanding of the efficiency of each parameter, especially in the case of cooperation, is still required to guide the integration of the two parameters in artificial medical systems. For example, the immobilization or encapsulation methods, the concentration and ratio, and the distribution, i.e., spatial relationship should be optimized in biomaterials and cell therapeutics. Overall, engineering the local delivery of integrin ligands and growth factors provides powerful and effective methods to regulate the stem cell fate.
